# Correlation Between Morphometric Measurements and Carrying Angle of Human Elbow

**DOI:** 10.7759/cureus.27331

**Published:** 2022-07-27

**Authors:** Vikas Verma, Arpit Singh, Narendra Singh Kushwaha, Yashwardhan Sharma, Ajai Singh

**Affiliations:** 1 Paediatric Orthopaedics, King George's Medical University, Lucknow, IND; 2 Orthopaedics, King George's Medical University, Lucknow, IND; 3 Orthopaedic Surgery, King George's Medical University, Lucknow, IND

**Keywords:** children's, paediatric orthopedics, elbow carrying angle, pearson correlation, morphometric

## Abstract

Purpose: This prospective cohort study aims to determine the correlation between morphometric measurements and the carrying angle of human elbow.

Methods: One hundred forty children were enrolled in the study. They were evaluated for age, sex, morphometric measurements, clinical carrying angle (CCA) and radiological carrying angle (RCA). The morphometric measurements included in the study were length of arms and forearms, inter-epicondylar distance of both sides, trans-trochanteric distance, height and body mass index. The mean of carrying angles in unrelated groups (gender and secondary sexual features) was compared using the unpaired t-test. Pearson’s correlation coefficient was calculated to determine the strength and direction of the relationship between carrying angle and continuous variables (age, height, body mass index, forearm length, arm length, inter-epicondylar distance and trans-trochanteric distance).

Results: The mean age was 5.84±4.76 years. Ninety-eight (70%) were males, and forty-two (30%) were females. The means of RCAs of the left side and right side were 9.07±2.13 and 8.85±2.09, respectively. The mean values of CCA on the left side and right side were 8.77±2.03 and 8.55±2.01 each. A significant positive correlation was found between CCA and age, weight, height, arm length, forearm length, inter-epicondylar distance and trans-trochanteric distance. CCA was found to be significantly negatively correlated with body mass index.

Conclusion: CCA is significantly correlated with age, BMI and morphometric measurements.

## Introduction

The angle formed between the forearm and arm, when the human elbow is fully extended and supinated, is known as the cubital angle or carrying angle [1]. It ranges from 0° to 25°. The range is 0°-21° in males and 0°-26° in females. A relatively distal location of the trochlea compared to the capitellum [2] and valgus angulation of the trochlea [3] are the anatomical explanation for the occurrence of carrying angle in the human elbow. A number of factors are well known to be associated with carrying angle. These include hand dominance [3], constitution [3], age [4,5], sex [5] and race [6]. However, the relationship between carrying angle and morphometric characteristics has not been extensively studied in paediatric population. The objective of this was to determine the correlation between carrying angle and morphometric factors, namely trans-trochanteric diameter, height, body mass index, length of a forearm, length of the arm and inter-epicondylar distance.

## Materials and methods

This cross-sectional study enrolled children up to 15 years of age presenting to the Outpatient Department of the Department of Paediatric Orthopaedics, King George's Medical University (KGMU). The exclusion criteria were congenital deformities, ligamentous laxity, abnormal muscle tone (motor neuron disease, head injury, cerebral palsy, paralysis due to any cause), metabolic bone diseases (Osteomalacia, Rickets), bone tumours, fractures, infection of bone or joints, burns or surgery to any of the upper extremities and history of trauma to any of the upper extremities.

Enrolment in the study was subject to consent. Written informed consent was obtained from one of the parents of the children. In children above the age of seven years, assent was obtained from the child in addition to the written informed consent by one of the parents. The study was approved by the ethics committee of KGMU vide letter number 402/Ethics/2020 dated December 10, 2020.

Enrolled children were evaluated for age, sex, morphometric measurements clinical carrying angle (CCA) and radiological carrying angle (RCA). The morphometric measurements included in the study were length of arms and forearms, inter-epicondylar distances of both sides, trans-trochanteric distance, height and body mass index. The sample size was estimated to be 140 using the formula

N= k[(Zα + Zβ)^2^(s1^2^ + s2^2^)]/d^2^

s1 = 3.95, s2 = 3.37 (where s1 and s2 are the maximum and minimum SD within groups); k = 4, the design effect taking into account the number of factors correlate [7]; d = (s1, s2), the minimum mean difference taken to be significant clinically; type I error was kept at 5%; type II error kept at 10%.

A standard pelvimeter was used to measure the trans-trochanteric diameter in a standing position [4]. The inter-epicondylar distance was measured using the technique described by Allouh [6]. The technique requires the arm to be elevated to the level of the shoulder and the elbow to be flexed to 90 degrees. This manoeuver is essential to increase the prominence of the epicondyles of the humerus, making them comfortably appreciable on palpation. We used a Vernier caliper to measure the distance between the two epicondyles (inter-epicondylar distance). We laid down the fixed lower jaw of the Vernier caliper on the lateral epicondyle and then moved the mobile lower jaw to touch the medial epicondyle. The length of the forearm was estimated by measuring the distance from the mid-point of a line joining the styloid processes of radius and ulna with the tip of the lateral epicondyle of the humerus.

The RCA was measured on an antero-posterior view Scanogram of the upper limb. Two central points on the humerus were joined to mark the humoral axis [8]. Two central points on the ulna were joined to mark the axis of the ulna [8]. A manual goniometer was used to measure the CCA. CCA was taken as the angle between the central axes of the arm and forearm. A line was drawn to connect to two epicondyles. Another line was drawn to connect the styloid processes of radius and ulna. The midpoints of these two lines were joined by a third line to mark the central axis of the forearm. The tip of acromion process was marked. A line was drawn to connect the tip of the acromion process with the midpoint of the inter-epicondylar line. This was taken as the central axis of the arm. The angle between these lines was taken as the CCA.

A password-protected computer was used to store data. Microsoft Excel sheet was used to record data. Statistical analysis was done using SPSS. Frequency tables were used to describe the categorical variables. Measures of central tendency (mean, median and mode) and measures of dispersions (standard deviation) were used to describe the continuous variables.

Means of carrying angles in unrelated groups (males versus females; those with secondary sexual characteristics versus those without secondary sexual characteristics) were compared using the Unpaired “t” test. The strength and direction of the relationship between continuous variables (age, length of forearm, length of arm, inter-epicondylar distance, trans-trochanteric distance, height and body mass index) and carrying angles were calculated using the Pearson’s correlation coefficient.

## Results

One hundred sixty subjects met the enrolment criteria of the study. Parents of 20 subjects did not consent to be included in the study. Therefore, a total of 140 subjects were enrolled. Ninety-eight (70%) were males and 42 (30%) were females. The mean age was 5.84±4.76 years. One hundred thirteen (80.7%) were less than 10 years of age and 27 (19.3%) were ≥ 10-15 years of age. The means of CCA in males and females were 8.61±2.01 and 8.94±2.01, respectively. The difference was statistically insignificant (p=0.641).

The baseline characteristics of the enrolled subjects are shown in Table 1. The mean values of CCA on the left side and right side were 8.77±2.03 and 8.55±2.01 each. Statistically, the difference between the mean values of CCAs on the two sides was insignificant (p=0.674). The means of RCAs on the left side and right side were 9.07±2.13 and 8.85±2.09, respectively. Statistically, the difference between the mean values of RCAs on the two sides was insignificant (p=0.654). Since the difference between the mean carrying angle (CCA and RCA) of the two sides was statistically insignificant, we computed the average mean carrying angle (CCA and RCA).

**Table 1:** Baseline characteristics of the enrolled subjects CCA - clinical carrying angle, RCA - radiological carrying angle

Variable name	Mean	SD	Median	Min	Max
Age (years)	5.84	4.76	6.00	.04	15.00
Height (cm)	104.31	29.61	106.00	58.00	152.00
Body Mass Index	17.17	2.88	16.91	11.62	28.40
Mean length of arm (cms)	18.38	5.66	19.96	9.10	29.82
Mean length of forearm (cms)	15.66	5.52	16.82	7.00	25.82
Mean inter-epicondylar distance(cms)	5.79	1.62	6.42	2.81	8.11
Transtrochanteric distance (cms)	28.48	7.55	30.42	13.21	40.81
CCA	8.66	2.02	8.30	5.80	13.40
RCA	8.99	2.11	8.50	5.90	13.60

**Figure 1:**
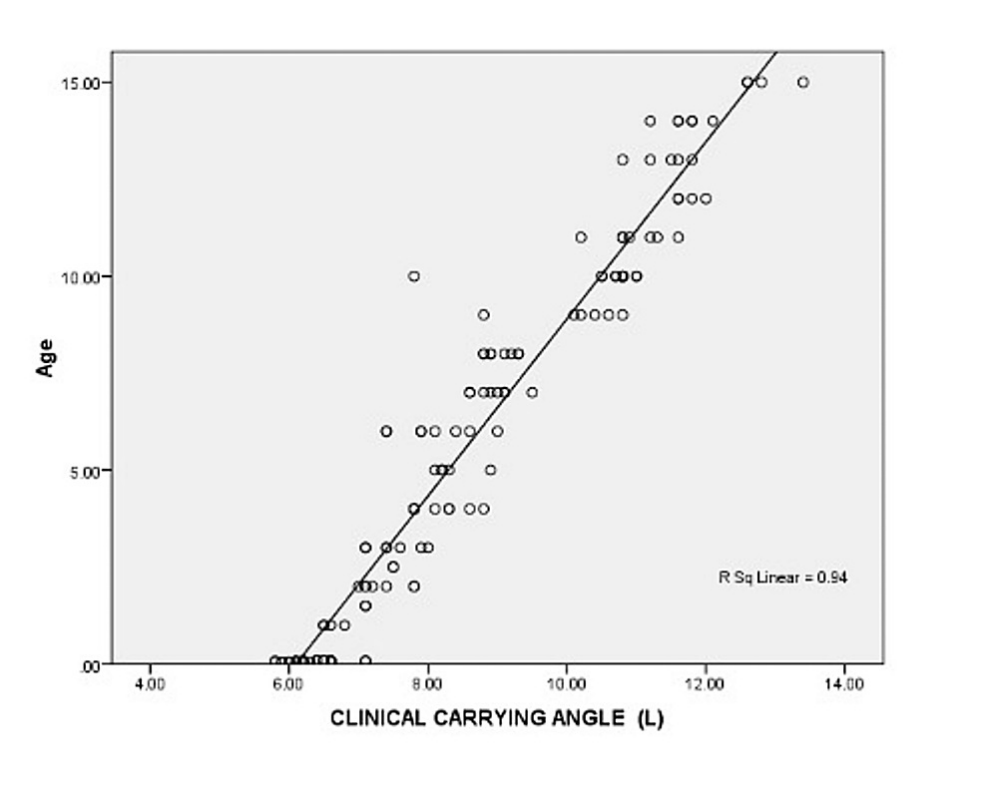
Correlation between age and carrying angle

Mean CCA and mean RCA were computed to be 8.66±2.02 and 8.99±2.11, respectively. The difference between the mean values of CCA and RCA was found to be statistically insignificant (p=0.624). Average values of morphometric measures (length of arm, length, of forearm, inter-epicondylar distance and trans-trochanteric distance) were computed by using measurements obtained for the two sides. Average values of the morphometric measures were used for correlation analysis to find the direction and strength of the relationship between carrying angle and morphometric measures. Since the means of CCA and RCA were not different, correlation analysis was done exclusively for CCA. Figure 1 shows the correlation between age and carrying angle.

**Figure 2:**
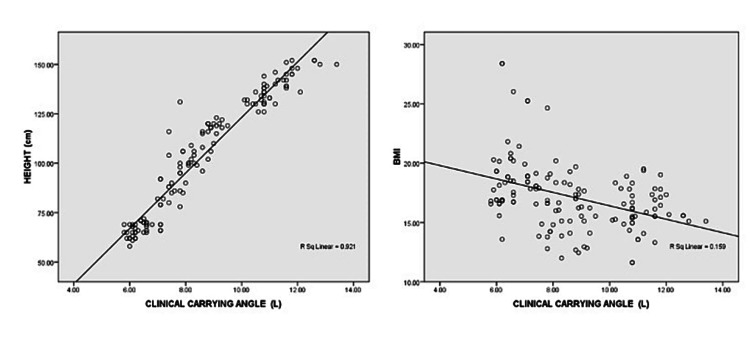
Correlation of CCA with height and BMI CCA - clinical carrying angle

CCA was found to be positively related to height and negatively correlated with BMI (Figure 2).

**Figure 3:**
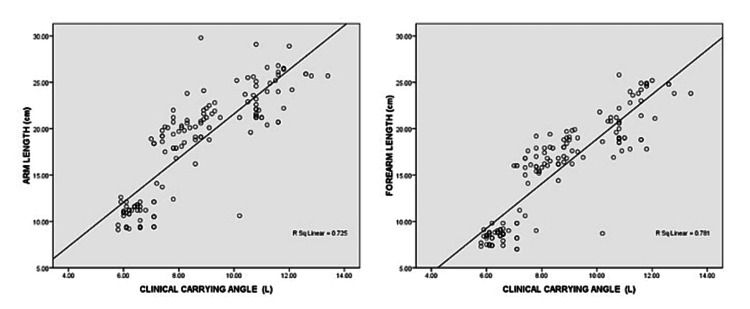
Correlation of CCA with arm length and forearm length CCA - clinical carrying angle

CCA was found to be positively correlated with arm length and forearm length (Figure 3).

**Figure 4:**
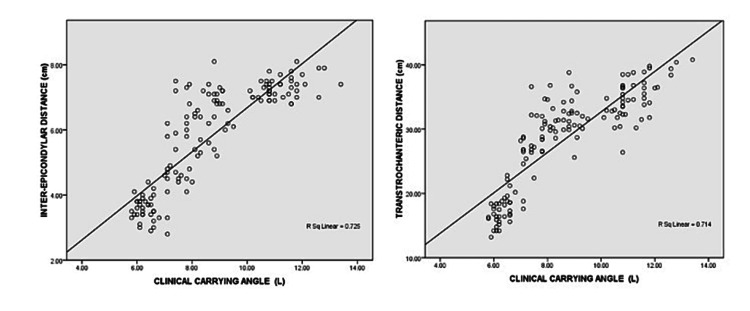
Correlation of CCA with inter-epicondylar distance and trans-trochanteric distance CCA - clinical carrying angle

CCA was found to be positively correlated with inter-epicondylar distance and trans-trochanteric distance (Figure 4).

A significant positive correlation was found between CCA and age, weight, height, arm length, forearm length, inter-epicondylar distance and trans-trochanteric distance. CCA was found to be significantly negatively correlated with BMI (Table 2).

**Table 2:** Correlation of clinical carrying angle with age, BMI and anthropometric measurements

	Clinical carrying angle	
	Pearson Correlation	P value
Clinical carrying angle	1	
Age	.970	<0.001
Height	.959	<0.001
BMI	-.398	0.159
Average Arm Length	.852	<0.001
Average Forearm length	.885	<0.001
Average Inter-epicondylar distance	.853	<0.001
Trans-trochanteric distance	0.846	<0.001

## Discussion

The present study confirms a significant positive correlation between CCA and increasing age. CCA has been reported in the literature to progressively increase till puberty when it reaches its maximum value [4,5]. There are conflicting reports in the literature regarding the age at which the carrying angle stabilizes. A study conducted on South Indian children reported a slight decrease after the age of 15 years. In contrast, a study on Brazillian children [9], reported that the carrying angle continued to increase up to the age of 16 years, after which it stabilized. The difference could be because of the different ethnicities of the enrolled subjects. According to some authors, the increase in carrying angle with age is related to gender and is greater in females than males [5,10]. Since we did not do a subgroup analysis, we cannot comment on the role played by gender on the relationship between age and carrying angle. However, a study by Beals [8] reported that the relationship between carrying angle and age is not influenced by gender. He hypothesized that the apparent difference in carrying angle between sexes may be due to greater ligamentous laxity in females compared to males. We excluded the individuals with ligamentous laxity, to prevent our results being biased by this particular clinical characteristic. We also took the precaution of comparing clinical and RCAs.

A higher carrying angle has been reported on the dominant side [3,10]. In children, a clear-cut hand dominance develops between the ages of 4-6 [11]. In order to investigate the effect of hand dominance on carrying angle, we would have had to exclude children below the age of six years. While it is a limitation of our study, we felt excluding children below the age of six years would be tantamount to missing a lot of vital information on the correlation of morphometric factors with carrying angle.

 A small number of studies have tried to correlate arm and forearm length with carrying angle. However, there is no definitive answer as to whether a correlation exists between these morphometric measures and carrying angle. A study by Balasubramaniam et al. that enrolled rural children from South India did not find a significant correlation between carrying angle and either arm or forearm [5]. However, a study by Kothapalli et al. that enrolled MBBS students reported a negative correlation between carrying angle and length of arm as well as that of the forearm [12]. We found a significant positive correlation between CCA and lengths of the arm and forearm. The present study as well as the study by Balasubramaniam et al. enrolled children while the study by Kothapalli et al. enrolled adults. Could age be the reason for this difference in the findings? We suggest a prediction modeling study employing regression analysis to provide a definitive answer to this question.

It is postulated that a higher carrying angle in females compared to males develops as a response to a proportionately broader pelvis implying that a greater carrying angle allows the forearm to clear the hips as the arm swings across the pelvis while walking. However, there is no definitive answer to the question regarding the strength and direction of the relationship between the value of carrying angle and pelvic diameter. We have found a significant positive correlation between CCA and trans-trochanteric diameter. An inverse correlation has been reported in the literature between carrying angle and trans-trochanteric distance [3]. However, a positive correlation between carrying angle and hip circumference has also been reported in the literature [13]. The notion that carrying angle helps the forearm clear the pelvis while walking seems to be faulty. The carrying angle forms when the elbow is supinated and extended. However, when a person walks, the elbow is held in a mid-prone and partially flexed position [14].

The present study has reported a significant positive correlation between height and CCA. The evidence in the literature regarding the strength and direction of the relationship between height and CCA is inconclusive. An inverse relationship between height and carrying angle has been reported in the literature [9,14]. Ruparelia et al. and Terra et al. postulated that in the case of a shorter person, the proximal end of a shorter ulna has to angulate more so as to bring the forearm in a pronated position for routine work [9,14]. This greater pronation in turn leads to the medial part of the ulna’s trochlear notch to go farther apart from the medial flange of the humeral trochlea leading to a higher carrying angle [9,14]. In contrast to the findings reported by Ruparelia et al. and Terra et al., a study by Sharma et al. reported a positive correlation on the left side but a negative correlation on the right side [7]. Our study as well as the studies by Ruparelia et al., Terra et al. and Sharma et al. focused on finding the correlation between the CCA and height. A definite answer to this question may be provided by a study that works out the association between CCA and height using regression modeling.

Evidence in the literature about the relationship between BMI and carrying angle is inconclusive. Studies have reported a positive correlation [15,16], and no correlation between BMI and carrying angle [4]. In the present study, the general trend was that of a decrease in the CCA as the BMI increased. However, the correlation was statistically insignificant (p=0.159). The difference may be explained based on the different ethnicities of the study population.

A negative correlation between carrying angle and inter-epicondylar distance was reported in a study, which compared the differences in carrying angles of Malaysian and Jordanian children [6]. The present study has reported a s a statistically significant positive correlation between carrying angle and the inter-epicondylar distance. The difference may be a result of the different ethnicities of the enrolled population. The ossification centre for trochlea appears much earlier than those for the capitulum and medial epicondyle. It is postulated that, during the process of ossification, the capitulum grows medially, which in turn leads to little room for the trochlear epiphysis [17]. Once the trochlear epiphysis starts ossifying, the space constraint medially causes it to grow more distally, resulting in the formation of the carrying angle [3].

Carrying angle has been reported to be greater in females compared to males in children [5] as well as adults [18,19]. However, the difference appears at the age of nine years and continues till stabilization [9]. In contrast, studies have also reported no difference irrespective of sex and age group [20,21]. A higher carrying angle in females than males is considered to be a secondary sexual characteristic [18]. A large majority of subjects in our cohort were children below the age of 10 years (81.1%), which may be the reason for the lack of significant difference between the carrying angle of males and females in the present study.

Limitations of the study

A limitation of our study is the lack of subgroup analysis based on sex. Hormonal factors may have a role to play in determining the correlation between carrying angle and studied variables. We advocate for a future study to investigate the factors associated with CCA in females and males. We have not investigated the effect of hand dominance on carrying angle as we did not want to exclude children below six years of age, and therefore we are not able to comment on the relationship between CCA and hand dominance.

Last but not the least, we have investigated the correlation of CCA with the studied variables. Correlation is not causation and the results reported by us may be influenced by confounding.

## Conclusions

The results of this study are consistent with other studies which have demonstrated a positive correlation of CCA with age of the child. While we have reported a significant positive correlation of CCA with height, arm length, forearm length, inter-epicondylar distance and trans-trochanteric distance, other studies have reported results ranging from a negative correlation to a positive correlation.

Whether morphometric measurements are truly associated with CCA is still a matter of conjecture, which may be resolved by a study using multivariable analysis of all factors reported to be associated with CCA.
